# The Combination of Salicylic Acid, Nicotinamide, and Proline Mitigates the Damage Caused by Salt Stress in Nasturtium (*Tropaeolum majus*)

**DOI:** 10.3390/plants14081156

**Published:** 2025-04-08

**Authors:** Thainan Sipriano dos Santos, Marcos Roberto Santos Correia, Luma Santos Sena, Laura Pereira dos Santos Santana, Geovanna Buique Gualberto da Silva, Keilane Silva Lima, Elienay Vinícius da Silva Dutra, Myriam El Adas, Maria Carolina Borges de Oliveira Ribeiro, João Everthon da Silva Ribeiro, Rogério Ferreira Ribas, Elania Freire da Silva, Alfredo Emilio Rubio-Casal, Aurélio Paes Barros Júnior, Xuguang Tang, Thieres George Freire da Silva, Alexandre Maniçoba da Rosa Ferraz Jardim, Toshik Iarley da Silva

**Affiliations:** 1 Center for Agrarian, Environmental, and Biological Sciences, Universidade Federal do Recôncavo da Bahia, Cruz das Almas 44380-000, BA, Brazil; thainansipriano96@gmail.com (T.S.d.S.); marcos_roberto9974@hotmail.com (M.R.S.C.); sennaluma@gmail.com (L.S.S.); laurapereira@aluno.ufrb.edu.br (L.P.d.S.S.); nanabuique@gmail.com (G.B.G.d.S.); keylalima1802@gmail.com (K.S.L.); elienayv@gmail.com (E.V.d.S.D.); myriam.eladas@aluno.ufrb.edu.br (M.E.A.); mariaborgesor@gmail.com (M.C.B.d.O.R.); ribas@ufrb.edu.br (R.F.R.); 2 Department of Agricultural and Forestry Sciences, Federal Rural University of the Semi-Arid, Mossoró 59625-900, RN, Brazil; j.everthon@hotmail.com (J.E.d.S.R.); elania.silva@alunos.ufersa.edu.br (E.F.d.S.); aurelio.barros@ufersa.edu.br (A.P.B.J.); 3 Department of Plant Biology and Ecology, University of Seville, Av. Reina Mercedes, s/n, 41012 Sevilla, Spain; aerubio@us.es; 4 Institute of Remote Sensing and Geosciences, Hangzhou Normal University, Hangzhou 311121, China; xgtang@hznu.edu.cn; 5 Department of Agricultural Engineering, Federal Rural University of Pernambuco, Dom Manoel de Medeiros Avenue, s/n, Dois Irmãos, Recife 52171-900, PE, Brazil; thieres.silva@ufrpe.br; 6 Department of Biodiversity, Institute of Biosciences, São Paulo State University—UNESP, Rio Claro 13506-900, SP, Brazil

**Keywords:** abiotic stress, edible flowers, plant hormones, vitamin B3, amino acids, gas exchange, inorganic and organic solutes

## Abstract

Salinity represents a significant challenge for agriculture, especially in semi-arid regions, affecting the growth and productivity of plants such as nasturtium (*Tropaeolum majus*), which is valued for its ornamental, medicinal, and food uses. Salt stress disrupts biochemical, physiological, and anatomical processes, limiting plant development. This study investigated the application of attenuators, including salicylic acid, nicotinamide, and proline, to mitigate the effects of salt stress on nasturtium cultivated in a hydroponic system. The treatments involved different combinations of these compounds under saline conditions (40 mM NaCl). The attenuators reduced the negative impacts of salt stress, promoting improvements in gas exchange, such as increased net photosynthesis, water-use efficiency, and stomatal conductance. Additionally, the treatments enhanced vegetative and reproductive growth, increasing the dry biomass of leaves, stems, and flowers, as well as the number of flowers and flower buds. The combination of salicylic acid, nicotinamide, and proline stood out by providing greater efficiency in carbon assimilation, stability of photosynthetic pigments, and higher tolerance to salt stress. These findings reinforce the potential of using attenuators to optimize the cultivation of nasturtium in saline environments, promoting higher productivity and plant quality.

## 1. Introduction

Nasturtium (*Tropaeolum majus* L., Tropaeolaceae), also known in Brazil as “capuchinha”, “nastúrtio”, and “chaguinha”, is a non-conventional food plant, grown as an ornamental, medicinal, and edible plant, and widely cultivated worldwide due to its adaptability and diverse applications [1]. Its flowers exhibit a distinctive peppery flavor, a hallmark of the species, which has made them one of the most commercially valued edible flowers globally [2]. The leaves and flowers of nasturtium are highly versatile, frequently used in salads, sandwiches, and as decorative elements in dishes and desserts, enhancing both the aesthetic appeal and functional value of culinary creations [3]. The plant is characterized by rounded leaves, vibrant flowers in shades of yellow, orange, and red, often adorned with dark internal markings, and greenish fruits. All parts of the plant are considered edible, with the exception of the roots [4]. Furthermore, nasturtium is rich in fatty acids, glucosinolates, flavonoids, and tetracyclic triterpenes, and in Brazil it is widely employed for the treatment of ulcers, colds, cellulitis, and anemia [5,6].

The quality of nasturtium plants is strongly influenced by environmental conditions [1]. Under abiotic stress, the plants often experience reduced growth and diminished visual quality of their flowers [7]. This impact is particularly pronounced in arid and semi-arid regions, where stressors such as drought and salinity are the primary limiting factors for the cultivation of edible flowers [6,8]. Under these conditions, water deficit and salinity disrupt essential physiological processes, such as photosynthesis, and impair root development, leading to reduced productivity and overall plant quality [2].

Salinity, characterized by the accumulation of soluble salts such as sodium (Na^+^) and chloride (Cl^−^), is a significant environmental challenge that threatens agricultural sustainability. In addition to adversely affecting crop growth and productivity, salinity degrades soil structure, reduces water infiltration, diminishes biodiversity, and contaminates water resources [9]. The use of high-salinity water exacerbates these effects by subjecting plants to combined osmotic and ionic stresses. These stresses lead to a marked decline in vegetative growth due to reduced water and nutrient uptake, structural cell disorganization, and excessive production of reactive oxygen species (ROS), which cause oxidative damage [10]. Soil salinity impacts approximately 800 million hectares globally, accounting for around 20% of irrigated agricultural land, particularly in arid, semi-arid, and coastal areas, due to poor irrigation and drainage practices, limited rainfall, high evaporation rates, and the use of saline water for irrigation [8,11]. Excess Na^+^ disrupts cellular metabolism and ionic balance, making it harmful to plants. For *T. majus*, salinity levels above 1 dS m^−1^ have been shown to inhibit rooting and seedling growth [12], although low salt concentrations may enhance biomass production [13].

In light of the challenges posed by soil salinization and increasing water scarcity, the adoption of sustainable agricultural practices is essential. In this context, hydroponics emerges as a promising alternative, offering greater water-use efficiency and enabling cultivation in regions with limited or low-quality water resources [14]. The exogenous application of stress alleviators has proven to be a promising strategy for mitigating the deleterious effects of salinity stress in nasturtium plants [6,8,15,16,17,18]. These compounds play a critical role in modulating various physiological and biochemical processes, enhancing the plants’ tolerance to adverse salinity conditions [19]. Among the most commonly used alleviators are phytohormones, vitamins, and amino acids, whose positive effects are widely documented in the literature.

Salicylic acid (SA) is a well-studied phytohormone with proven effects in mitigating damage caused by salinity stress [17]. Its exogenous application reduces oxidative stress, regulates ion uptake, promotes water balance, and positively influences stomatal behavior, gas exchange, chlorophyll fluorescence, and osmoregulation [20]. Nicotinamide (nicotinamide or niacin) functions as a growth regulator, influencing physiological processes such as enzyme, nucleic acid, and protein biosynthesis, while also serving as a critical coenzyme [21]. Proline, an amino acid involved in primary metabolism, plays a central role under stress conditions [18]. As one of the most accumulated compatible solutes in plants exposed to salinity, water deficit, or extreme temperatures, proline contributes to cellular protection and the maintenance of metabolic processes [22].

The innovative aspects of this research lie in its pioneering exploration of the combined use of salicylic acid, proline, and nicotinamide to mitigate salinity-induced damage in hydroponically grown nasturtium plants. Unlike previous studies that have focused individually on these compounds, this study evaluates their synergistic effects on physiological and biochemical processes, including gas exchange, photosynthetic pigments, organic and inorganic solutes, and overall plant growth. The novelty also extends to assessing the viability of hydroponic cultivation as a sustainable alternative for growing nasturtium under saline conditions, providing valuable insights into enhancing plant tolerance and productivity in challenging environments.

The selection of SA, nicotinamide, and proline as stress alleviators was based on their complementary roles in enhancing plant resilience under salt stress through distinct mechanisms. SA is known for reducing oxidative stress, regulating ion uptake, improving water balance, and enhancing photosynthetic efficiency. Nicotinamide acts as a growth regulator, influencing enzyme, nucleic acid, and protein biosynthesis, while also serving as a crucial coenzyme. Proline functions as an osmoprotectant, contributing to osmotic balance, membrane stabilization, and ROS scavenging. Their combined application offers a synergistic effect, addressing multiple stress factors simultaneously, which is particularly relevant under saline conditions where plants face osmotic, ionic, and oxidative stress. Additionally, while these alleviators have been individually studied, their joint application in hydroponically grown nasturtium under salinity conditions is an innovative approach that aims to enhance plant tolerance and productivity more effectively than individual application.

Although progress has been made in understanding the physiological and biochemical responses of nasturtium to salt stress, significant gaps remain, particularly concerning the use of attenuators such as salicylic acid, nicotinamide, and proline. These compounds have shown promising potential in mitigating the negative effects of salinity by regulating ion uptake, maintaining water balance, stabilizing membranes, modulating antioxidant metabolism, and improving photosynthetic efficiency. However, studies so far have mainly focused on the isolated application of these attenuators, without adequately exploring the combined effects that could provide a more robust approach to enhancing nasturtium’s tolerance to salt stress. Investigating the joint use of these compounds in hydroponic systems exposed to salinity represents an innovative strategy that could significantly improve the productivity and quality of nasturtium grown under adverse conditions.

Despite advances in understanding the role of stress alleviators, studies on their use in nasturtium plants cultivated under salinity stress in hydroponic systems remain scarce. This study is a pioneering effort in exploring the combined use of salicylic acid, proline, and nicotinamide to mitigate salinity-induced damage in hydroponically grown nasturtium plants. Accordingly, the present research aimed to evaluate the effects of salicylic acid, nicotinamide, and proline application on gas exchange, photosynthetic pigments, organic and inorganic solutes, and the growth of *Tropaeolum majus* cultivated in a hydroponic system under salinity conditions.

## 2. Results

### 2.1. Effect of Salt Stress on Gas Exchange

**Figure 1:**
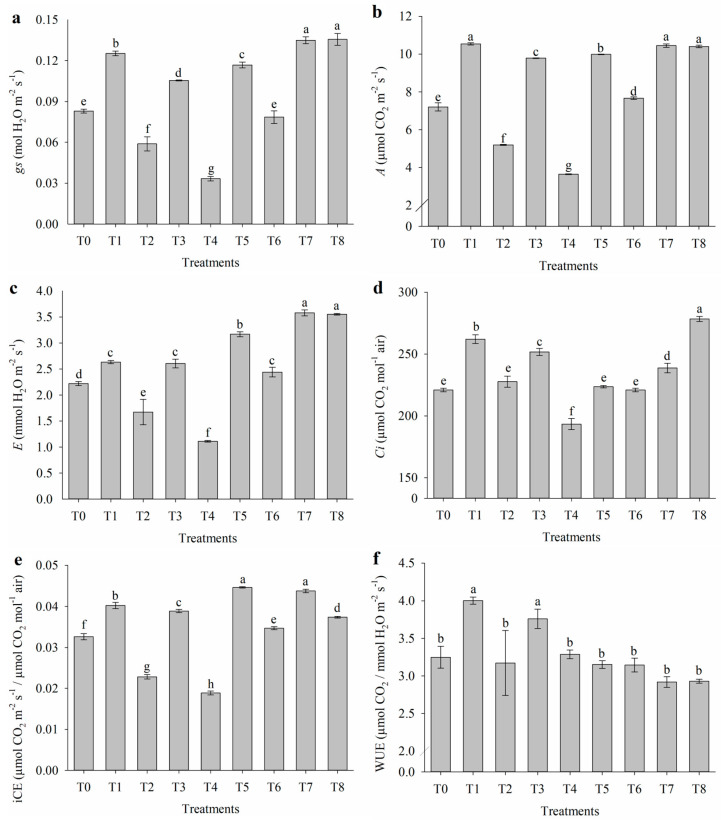

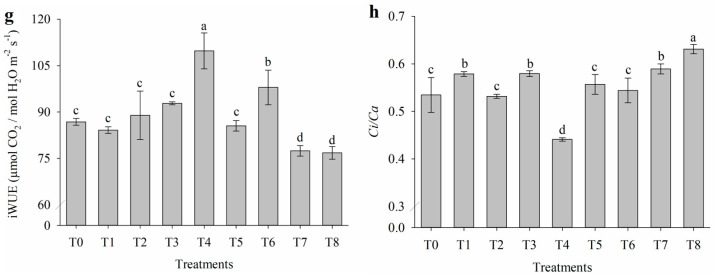
(**a**) Stomatal conductance (*gs*), (**b**) net photosynthesis (*A*), (**c**) transpiration rate (*E*), (**d**) intercellular CO_2_ concentration (*Ci*), (**e**) intrinsic carboxylation efficiency (iCE), (**f**) instantaneous water-use efficiency (WUE), (**g**) intrinsic water-use efficiency (iWUE), and (**h**) *Ci*/*Ca* ratio of *Tropaeolum majus* subjected to salt stress and the application of salicylic acid (SA), nicotinamide (NAM), and proline (Pro). T0 = control (0 mM NaCl); T1 = 40 mM NaCl; T2 = 40 mM NaCl + Pro; T3 = 40 mM NaCl + SA; T4 = 40 mM NaCl + NAM; T5 = 40 mM NaCl + Pro + SA; T6 = 40 mM NaCl + Pro + NAM; T7 = 40 mM NaCl + SA + NAM; and T8 = 40 mM NaCl + Pro + SA + NAM. Means followed by the same letter do not differ according to the Scott–Knott test (*p* ≤ 0.05). Bars are means ± standard error (*n* = 4).

Salt stress and the application of attenuators influenced gas exchange in *T. majus* (Figure 1). The isolated application of nicotinamide (T4) reduced stomatal conductance (*gs*; Figure 1a), net photosynthesis (*A*; Figure 1b), transpiration rate (*E*; Figure 1c), intercellular CO_2_ concentration (*Ci*; Figure 1d), intrinsic carboxylation efficiency (iCE; Figure 1e), and the ratio of intercellular to external CO_2_ concentration (*Ci*/*Ca*; Figure 1h). On the other hand, the combination of nicotinamide with salicylic acid (T7) and with salicylic acid and proline (T8) increased stomatal conductance (0.13 and 0.14 mol H_2_O m^−2^ s^−1^, respectively) and the transpiration rate (3.58 and 3.55 mmol H_2_O m^−2^ s^−1^, respectively). Additionally, the combined application of all three attenuators increased the intercellular CO_2_ concentration (278.33 µmol CO_2_ mol^−1^ air) and the *Ci*/*Ca* ratio (0.63), suggesting a possible improvement in the plant’s adaptive capacity under salt stress conditions.

Under salt stress, in the absence of attenuators (T1), and with the application of nicotinamide combined with salicylic acid (T7) and with all three attenuators (T8), an increase in net photosynthesis (*A*, Figure 1b) was observed, reaching 10.54, 10.15, and 10.40 µmol CO_2_ m^−2^ s^−1^, respectively. Intrinsic carboxylation efficiency (iCE) improved with the application of salicylic acid combined with proline (T5) and with nicotinamide (T7), both resulting in 0.044. Water-use efficiency (WUE; Figure 1f) was elevated under salinity without attenuators (T1) and with the application of salicylic acid (T3), with values of 4.00 and 3.76 µmol CO_2_/mmol H_2_O m^−2^ s^−1^, respectively. Additionally, intrinsic water-use efficiency (iWUE; Figure 1g) increased under salinity conditions with the application of nicotinamide (109.7 µmol CO_2_/mol H_2_O m^−2^ s^−1^).

### 2.2. Impact of Salt Stress on Photosynthetic Pigments

**Figure 2:**
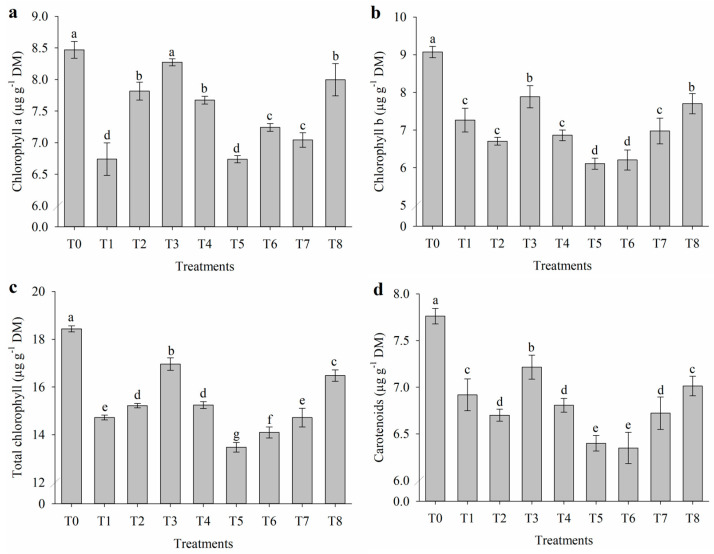
(**a**) Chlorophyll a, (**b**) chlorophyll b, (**c**) total chlorophyll, and (**d**) carotenoids of *Tropaeolum majus* subjected to salt stress and the application of salicylic acid (SA), nicotinamide (NAM), and proline (Pro). T0 = control (0 mM NaCl); T1 = 40 mM NaCl; T2 = 40 mM NaCl + Pro; T3 = 40 mM NaCl + SA; T4 = 40 mM NaCl + NAM; T5 = 40 mM NaCl + Pro + SA; T6 = 40 mM NaCl + Pro + NAM; T7 = 40 mM NaCl + SA + NAM; and T8 = 40 mM NaCl + Pro + SA + NAM. Means followed by the same letter do not differ according to the Scott–Knott test (*p* ≤ 0.05). Bars are means ± standard error (*n* = 4).

The application of attenuators to nasturtium plants cultivated under salinity conditions influenced the levels of photosynthetic pigments (Figure 2). Salt stress reduced the contents of chlorophyll a (Figure 2a), chlorophyll b (Figure 2b), total chlorophyll (Figure 2c), and carotenoids (Figure 2d). However, the application of salicylic acid (T3) and the combination of all three attenuators (T8) contributed to a more stable maintenance of chlorophyll indices under salinity. On the other hand, the combined application of proline with salicylic acid (T5) resulted in a reduction in chlorophyll a (6.73 µg g^−1^ dry mass), chlorophyll b (6.11 µg g^−1^ DM), total chlorophyll (13.48 µg g^−1^ DM), and carotenoids (6.40 µg g^−1^ DM). Additionally, the combination of proline with nicotinamide (T6) also reduced chlorophyll b (6.20 µg g^−1^ DM) and carotenoid levels (6.35 µg g^−1^ DM), reinforcing the negative effects of these interactions on photosynthetic pigments.

### 2.3. Inorganic Solutes of Leaves and Flowers

**Figure 3:**
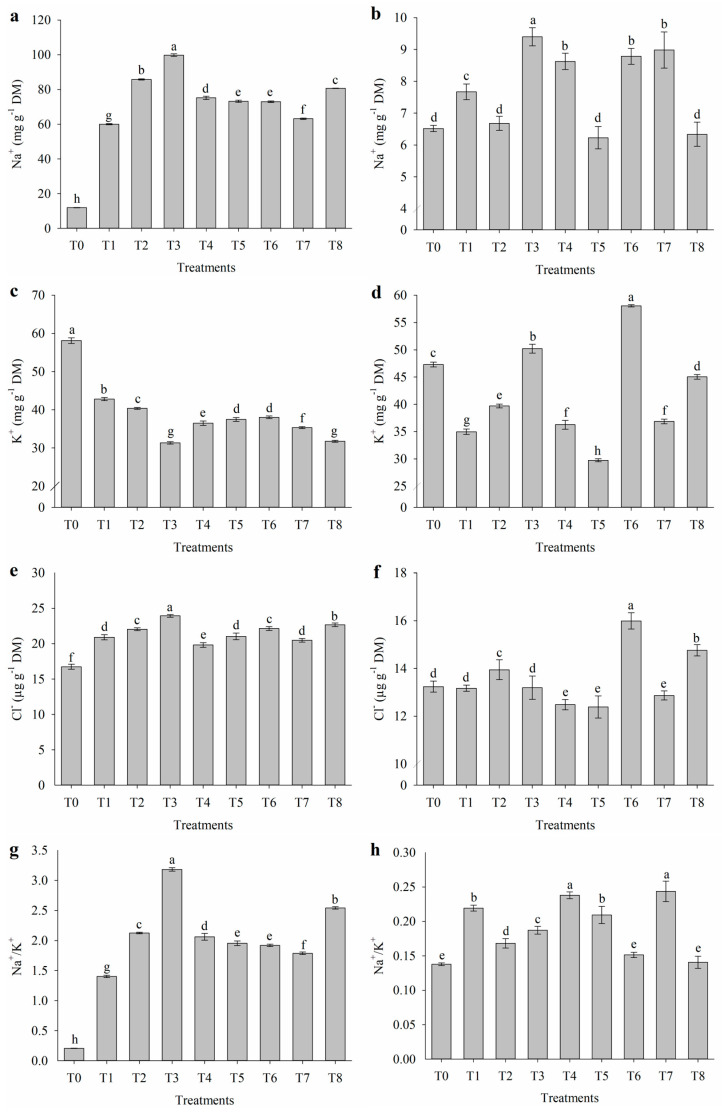
(**a**,**b**) Sodium (Na^+^), (**c**,**d**) potassium (K^+^), (**e**,**f**) chloride (Cl^−^), and (**g**,**h**) Na^+^/K^+^ ratio in leaves and flowers, respectively, of *Tropaeolum majus* subjected to salt stress and the application of salicylic acid (SA), nicotinamide (NAM), and proline (Pro). T0 = control (0 mM NaCl); T1 = 40 mM NaCl; T2 = 40 mM NaCl + Pro; T3 = 40 mM NaCl + SA; T4 = 40 mM NaCl + NAM; T5 = 40 mM NaCl + Pro + SA; T6 = 40 mM NaCl + Pro + NAM; T7 = 40 mM NaCl + SA + NAM; and T8 = 40 mM NaCl + Pro + SA + NAM. Means followed by the same letter do not differ according to the Scott–Knott test (*p* ≤ 0.05). Bars are means ± standard error (*n* = 4).

The application of salt stress attenuators influenced the levels of inorganic solutes in the leaves and flowers of nasturtium (Figure 3). Under salt stress conditions, there was an increase in Na^+^, Cl^−^, and the Na^+^/K^+^ ratio in the leaves (Figure 3a,e,g), differing from the control (T0). The treatment with salicylic acid (T3) resulted in the highest accumulation of these ions, with values of 99.8 mg g^−1^ DM, 24.0 µg g^−1^ DM, and 3.2, respectively. In the flowers, salt stress also led to an increase in Na^+^ levels (Figure 3b). The treatment with salicylic acid (T3) resulted in the highest Na^+^ accumulation in the flowers, reaching 9.4 mg g^−1^ DM. Conversely, the application of proline alone (T2) and its combinations with salicylic acid (T5) or with salicylic acid and nicotinamide (T8) maintained Na^+^ levels in the flowers similar to the control (T0), with values of 6.68, 6.23, 6.34, and 6.52 mg g^−1^ DM, respectively.

Under salt stress conditions, the application of salicylic acid alone (T3) and in combination with all three attenuators (T8) resulted in the lowest K^+^ levels in the leaves (Figure 3c), with values of 31.4 and 31.8 mg g^−1^ DM, respectively, while the control (T0) showed the highest accumulation, reaching 58.1 mg g^−1^ DM. Salinity also affected the levels of K^+^ and Cl^−^ in the flowers (Figure 3d,f). The combination of proline with nicotinamide (T6) significantly increased K^+^ and Cl^−^ levels, registering values of 58.1 mg g^−1^ DM and 16.0 µg g^−1^ DM, respectively. In contrast, the combination of proline with salicylic acid (T5) reduced K^+^ accumulation in the flowers, with a value of 29.7 mg g^−1^ DM. Additionally, the application of nicotinamide alone (T4), combined with salicylic acid (T7), and the combination of proline with salicylic acid (T5) reduced Cl^−^ levels in the flowers, showing values of 12.5, 12.9, and 12.4 µg g^−1^ DM, respectively. Regarding the Na^+^/K^+^ ratio in the leaves (Figure 3h), the combinations of proline with nicotinamide (T6) and all three attenuators (T8) under salt stress resulted in the lowest values (0.15, 0.14, and 0.14, respectively), similar to the control (T0). Conversely, nicotinamide alone (T4) and combined with salicylic acid (T7) showed the highest Na^+^/K^+^ ratios, with values of 0.23 and 0.24, respectively.

### 2.4. Organic Solutes of Leaves and Flowers

**Figure 4:**
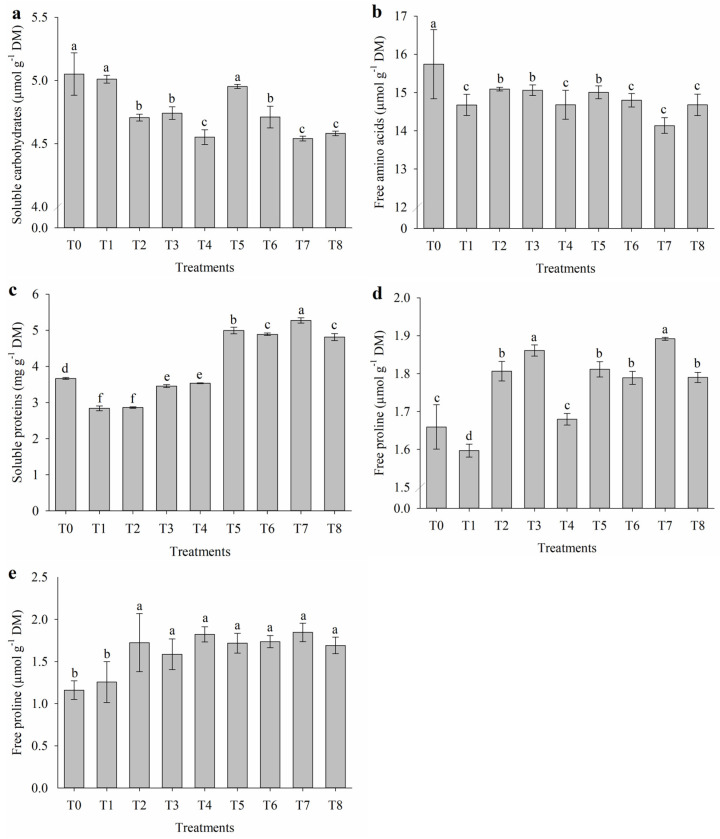
(**a**) Soluble carbohydrates, (**b**) free amino acids, (**c**) soluble proteins, (**d**) free proline in flowers, and (**e**) free proline in leaves of *Tropaeolum majus* subjected to salt stress and the application of salicylic acid (SA), nicotinamide (NAM), and proline (Pro). T0 = control (0 mM NaCl); T1 = 40 mM NaCl; T2 = 40 mM NaCl + Pro; T3 = 40 mM NaCl + SA; T4 = 40 mM NaCl + NAM; T5 = 40 mM NaCl + Pro + SA; T6 = 40 mM NaCl + Pro + NAM; T7 = 40 mM NaCl + SA + NAM; and T8 = 40 mM NaCl + Pro + SA + NAM. Means followed by the same letter do not differ according to the Scott–Knott test (*p* ≤ 0.05). Bars are means ± standard error (*n* = 4).

Under salt stress conditions, the application of attenuators influenced the levels of organic solutes in nasturtium flowers (Figure 4). The levels of soluble carbohydrates, free amino acids, and soluble proteins in the leaves were not affected by the treatments. Soluble carbohydrate levels (Figure 4a) were reduced by the application of nicotinamide alone (T4), in combination with salicylic acid (T7), and in the interaction among all three attenuators (T8), showing values of 4.54, 4.55, and 4.58 µmol g^−1^ DM, respectively. Conversely, the combination of proline with salicylic acid (T5) resulted in the highest levels of soluble carbohydrates, equivalent to those observed in the control (T0) and in the salt-stressed treatment without attenuators (T1), with values of 4.95, 5.01, and 5.00 µmol g^−1^ DM, respectively.

Salinity reduced the contents of free amino acids in the flowers of nasturtium (Figure 4b). The control (T0) showed the highest accumulation, with 15.74 µmol g^−1^ DM. Under salinity conditions, the highest contents of free amino acids were observed in treatments without attenuators (T1) and with the application of nicotinamide alone (T4), combined with proline (T6), salicylic acid (T7), and with both attenuators (T8), with values of 14.7 (T1), 14.7 (T4), 14.8 (T6), 14.1 (T7), and 14.7 µmol g^−1^ DM (T8), respectively. The application of nicotinamide combined with salicylic acid (T7) increased the levels of soluble proteins in the flowers, reaching 5.30 mg g^−1^ DM (Figure 4c). In contrast, under salinity without attenuators (T1) and with the application of proline alone (T2), the levels of soluble proteins decreased to 2.84 and 2.87 mg g^−1^ DM, respectively.

Free proline levels in the flowers (Figure 4d) were increased by the application of salicylic acid alone (T3) and in combination with nicotinamide (T7), reaching values of 1.87 and 1.89 µmol g^−1^ DM, respectively. On the other hand, the lowest free proline level was observed under salinity without attenuators (T1), with 1.60 µmol g^−1^ DM. Free proline levels in the leaves (Figure 4e) were also influenced by salinity and the application of attenuators. All treatments with attenuators increased free proline levels compared to the control (T0) and the salt-stressed treatment without attenuators (T1), which showed the lowest values at 1.16 and 1.25 µmol g^−1^ DM, respectively. However, the treatments with attenuators did not differ significantly from each other.

### 2.5. Growth and Biomass Production

**Figure 5:**
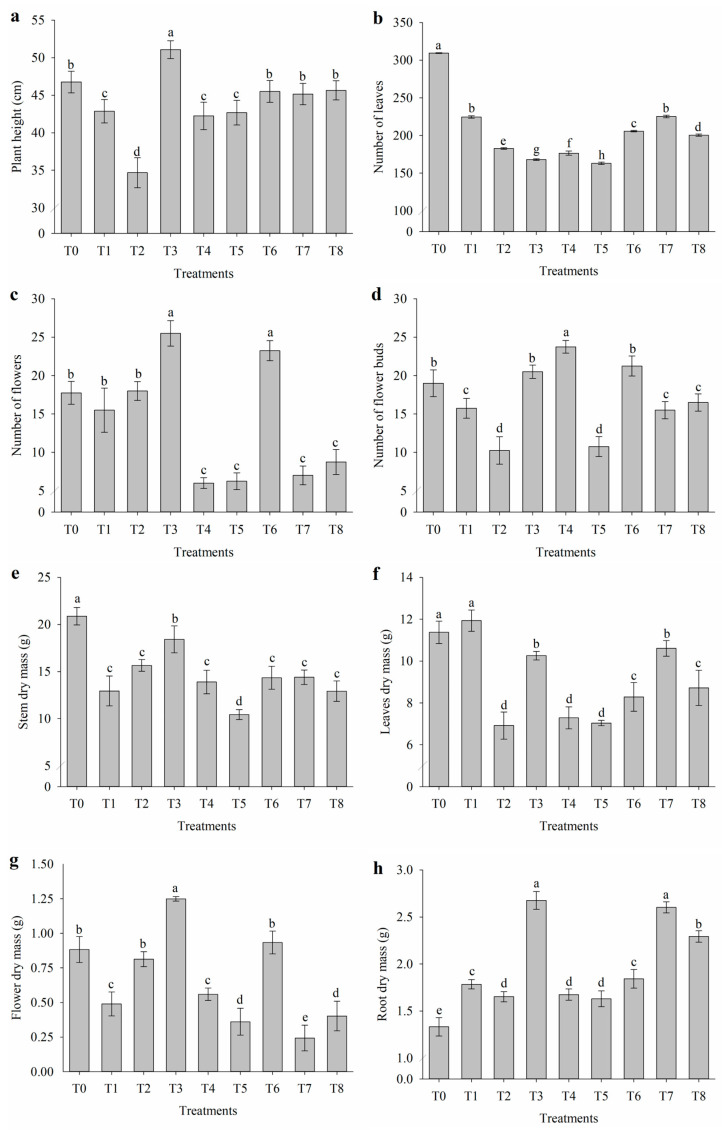
(**a**) Plant height, (**b**) number of leaves, (**c**) number of flowers, (**d**) number of flower buds, (**e**) stem dry mass, (**f**) leaves dry mass, (**g**) flower dry mass, and (**h**) root dry mass of *Tropaeolum majus* subjected to salt stress and the application of salicylic acid (SA), nicotinamide (NAM), and proline (Pro). T0 = control (0 mM NaCl); T1 = 40 mM NaCl; T2 = 40 mM NaCl + Pro; T3 = 40 mM NaCl + SA; T4 = 40 mM NaCl + NAM; T5 = 40 mM NaCl + Pro + SA; T6 = 40 mM NaCl + Pro + NAM; T7 = 40 mM NaCl + SA + NAM; and T8 = 40 mM NaCl + Pro + SA + NAM. Means followed by the same letter do not differ according to the Scott–Knott test (*p* ≤ 0.05). Bars are means ± standard error (*n* = 4).

The application of attenuators to nasturtium plants cultivated under salt stress influenced growth and biomass production (Figure 5). The tallest plants were observed with the application of salicylic acid (T3), reaching 51.1 cm, while the application of proline alone (T2) resulted in the shortest plants at 34.7 cm (Figure 5a). Salt stress reduced the number of leaves compared to the control, highlighting the negative impacts of this condition. However, the combined application of nicotinamide with salicylic acid (T7) resulted in the highest number of leaves (225.5) in salt-stressed plants, indicating a positive effect of this treatment in mitigating the damages caused by salinity (Figure 5b).

Salinity and the application of attenuators influenced the number of flowers in nasturtium (Figure 2c). The application of salicylic acid (T3) and the combination of proline with nicotinamide (T6) increased the number of flowers, recording values of 25.5 and 23.25, respectively. The number of flower buds (Figure 5d) was also affected, with the highest value observed in the treatment with nicotinamide alone (T4), which reached 23.75 under salt stress. Conversely, the application of proline alone (T2) and its combination with salicylic acid (T5) resulted in reductions in the number of flower buds, with values of 10.25 and 10.75, respectively.

The dry mass of the stems (Figure 5e), leaves (Figure 5f), and flowers (Figure 5g) was affected by salt stress and the application of attenuators. Under salinity conditions, the combination of proline with salicylic acid (T5) resulted in the greatest reduction in stem dry mass, recording 10.44 g. Conversely, the highest values of leaves dry mass were observed in the control (T0) and in the salt-stressed treatment without attenuators (T1), with 11.38 and 11.93 g, respectively. The application of proline alone (T2), combined with salicylic acid (T5), and nicotinamide alone (T4) reduced the leaves’ dry mass under salt stress, with values of 6.92, 7.04, and 7.29 g, respectively. Regarding flower dry mass, the application of salicylic acid alone (T3) resulted in a significant increase, reaching 1.25 g. In contrast, the application of nicotinamide combined with salicylic acid (T7) reduced the flower dry mass to 0.25 g. The application of salicylic acid (T3) and its combination with nicotinamide increased the root dry mass of nasturtium (Figure 5h).

## 3. Discussion

The application of attenuators represents an alternative to mitigate the harmful effects caused by salinity. Nicotinamide, when applied alone, inhibited stomatal conductance. However, when combined with other attenuators, its negative effects were possibly modulated. This is directly linked to CO_2_ uptake for photosynthesis, which consequently promotes better development of nasturtium plants. The results of this study provide novel insights into the physiological and biochemical responses of *T. majus* to salinity stress and the mitigating effects of exogenously applied stress alleviators. Salt stress is known to impair gas exchange parameters by disrupting stomatal conductance, photosynthetic efficiency, and water-use dynamics, which are critical for plant survival under adverse conditions [23].

The combined application of nicotinamide with salicylic acid (T7) and with salicylic acid and proline (T8) showed an improvement in *gs* and *E*, suggesting a synergistic effect between these compounds in modulating stomatal behavior. The enhanced transpiration rates observed in T7 and T8 indicate improved water regulation, which may aid in the dissipation of heat and the maintenance of cellular homeostasis under saline conditions [24].

The simultaneous application of nicotinamide, salicylic acid, and proline (T8) resulted in the highest intercellular CO_2_ concentration (*Ci*) and the greatest *Ci*/*Ca* ratio, indicating an enhanced ability of plants to maintain carbon assimilation under salt stress conditions. The increased *Ci*/*Ca* ratio suggests that the treatment promoted a balance between stomatal conductance (*gs*) and photosynthetic activity (*A*), reducing the limitations imposed by stomatal closure typically associated with salt stress [25]. Previous studies highlight that *Ci* acts as a central signal in the interaction between *gs* and the mesophyll, regulating stomatal responses and photosynthetic assimilation based on variables such as light intensity and CO_2_ availability [26]. Although the relationship between *gs* and *Ci* is not linear and depends on specific conditions, such as species and the intensity of photosynthetically active radiation (PAR) [27], the higher *Ci*/*Ca* observed in T8 suggests greater efficiency in CO_2_ uptake and utilization by the mesophyll, even in adverse environments. These results support the hypothesis that metabolic interventions with compounds such as nicotinamide, salicylic acid, and proline can mitigate the effects of salt stress, promoting physiological resilience and optimizing photosynthesis under high salinity conditions. The application of nicotinamide has previously been shown to improve gas exchange in maize grown under salt stress [28] and in pumpkin cultivated hydroponically [29].

The application of attenuators represents an alternative to mitigate the harmful effects caused by salinity. Nicotinamide, when applied alone, inhibited stomatal conductance. However, when combined with other attenuators, its negative effects were possibly modulated. This is directly linked to CO_2_ uptake for photosynthesis, which consequently promotes better development of nasturtium plants. The results of this study provide novel insights into the physiological and biochemical responses of nasturtium to salinity stress and the mitigating effects of exogenously applied stress alleviators. Salt stress is known to impair gas exchange parameters by disrupting stomatal conductance, photosynthetic efficiency, and water-use dynamics, which are critical for plant survival under adverse conditions. The combined application of nicotinamide with salicylic acid (T7) and with salicylic acid and proline (T8) showed an improvement in *gs* and *E*, suggesting a synergistic effect between these compounds in modulating stomatal behavior. The enhanced transpiration rates observed in T7 and T8 indicate improved water regulation, which may aid in the dissipation of heat and the maintenance of cellular homeostasis under saline conditions. Importantly, T8 exhibited the highest values of *gs* and *E*, indicating its greater efficiency in enhancing gas exchange and promoting physiological resilience compared to the other treatments.

The application of nicotinamide, salicylic acid, and proline (T8) resulted in the highest *Ci* and the greatest *Ci*/*Ca* ratio, indicating an enhanced ability of plants to maintain carbon assimilation under salt stress conditions. The increased *Ci*/*Ca* ratio suggests that the treatment promoted a balance between *gs* and *A*, reducing the limitations imposed by stomatal closure typically associated with salt stress. This greater efficiency in CO_2_ uptake and utilization by the mesophyll observed in T8 demonstrates its superiority over other treatments in enhancing photosynthetic performance under adverse environments.

Salt stress reduced the contents of chlorophyll a, chlorophyll b, total chlorophyll, and carotenoids, reflecting its deleterious effects on the photosynthetic apparatus. This reduction can be attributed to oxidative damage, chloroplast disorganization, and either the disruption of pigment biosynthesis or the acceleration of their degradation, which are common physiological responses to ionic and osmotic stress [30]. Among the treatments, salicylic acid (T3) and the combination of salicylic acid, proline, and nicotinamide (T8) demonstrated a stabilizing effect on chlorophyll levels under salinity. The ability of salicylic acid to mitigate damage to pigment levels aligns with its known role in enhancing antioxidant defense systems, reducing oxidative stress, and protecting chloroplast integrity [17]. The maintenance of pigment levels in T8 suggests a synergistic interaction among salicylic acid, proline, and nicotinamide, contributing to greater chloroplast stability and sustained photosynthetic activity under saline conditions.

Salt stress reduced the contents of chlorophyll a, chlorophyll b, total chlorophyll, and carotenoids, reflecting its deleterious effects on the photosynthetic apparatus. This reduction can be attributed to oxidative damage, chloroplast disorganization, and either the disruption of pigment biosynthesis or the acceleration of their degradation, which are common physiological responses to ionic and osmotic stress. Among the treatments, salicylic acid (T3) and the combination of salicylic acid, proline, and nicotinamide (T8) demonstrated a stabilizing effect on chlorophyll levels under salinity. The ability of T8 to maintain pigment levels suggests that its combination of attenuators provided superior protection to the photosynthetic machinery, enhancing chloroplast stability and sustaining photosynthetic activity more effectively than other treatments.

Salt stress in plants, primarily characterized by ionic stress due to excessive Na^+^ accumulation, leads to increased foliar contents of these ions, electrolyte leakage, ROS production, lipid peroxidation, and antioxidant enzyme activity. The balance between Na^+^, Cl^−^, and K^+^ in tissues is a critical factor for plant salinity tolerance [31]. Under saline conditions, increased levels of Na^+^, Cl^−^, and the Na^+^/K^+^ ratio were observed in leaves, indicating the accumulation of these ions as a response to osmotic and ionic stress.

The accumulation of Na^+^ in low amounts is a common strategy for plants to adjust osmotic potential under salinity. However, an increased Na^+^/K^+^ ratio reflects an ionic imbalance that can compromise crucial metabolic processes, leading to severe ionic stress in plants [32] and causing damage to physiological and biochemical processes and growth, as observed in this study. The treatment with salicylic acid alone (T3) resulted in the highest accumulation of Na^+^, Cl^−^, and Na^+^/K^+^ ratio in leaves, suggesting that in this case, salicylic acid may not have been sufficient to regulate ion transport and compartmentalization [33]. Conversely, the application of proline alone (T2), in combination with salicylic acid (T5), or with salicylic acid and nicotinamide (T8), maintained Na^+^ levels in flowers similar to the control (T0), indicating that these treatments were more effective in mitigating the effects of salt stress. Proline, recognized as a compatible solute, likely played a role in osmotic stabilization and cellular structure protection, minimizing excessive accumulation of toxic ions [34].

The contents of K^+^ exhibited distinct behavior between leaves and flowers. In leaves, treatments T3 (salicylic acid alone) and T8 (combination of all three attenuators) resulted in the lowest K^+^ levels, possibly due to ionic competition with Na^+^, which impairs K^+^ uptake and transport. The high molecular similarity between Na^+^ and K^+^ allows cytoplasmic Na^+^ accumulation, facilitated by competition in shared transport systems, to inhibit K^+^ uptake and replace K^+^ in K^+^-dependent enzymes. However, Na^+^ cannot perform the biological functions of K^+^ [35]. In flowers, the combination of proline with nicotinamide (T6) significantly increased K^+^ and Cl^−^ levels, suggesting that this combination may have promoted ionic redistribution and the maintenance of homeostasis in reproductive tissues, which are essential for plant development [36].

Regarding the Na^+^/K^+^ ratio in leaves, treatments such as T6 (proline with nicotinamide) and T8 (combination of all three attenuators) showed values similar to the control (T0), demonstrating greater efficiency in regulating ionic balance under saline conditions [37]. On the other hand, nicotinamide applied alone (T4) and combined with salicylic acid (T7) resulted in the highest Na^+^/K^+^ ratios, suggesting that these combinations may have limited the selectivity of ionic transport, favoring Na^+^ accumulation at the expense of K^+^ [33]. The contents of K^+^ exhibited distinct behavior between leaves and flowers. In leaves, treatments T3 (salicylic acid alone) and T8 (combination of all three attenuators) resulted in the lowest K^+^ levels, possibly due to ionic competition with Na^+^, which impairs K^+^ uptake and transport. However, the fact that T8 maintained a Na^+^/K^+^ ratio similar to the control (T0) highlights its superior ability to regulate ionic balance under saline conditions, promoting ionic homeostasis more effectively than other treatments.

Salinity is widely recognized for causing metabolic alterations in plants, particularly in the accumulation of osmotic solutes and the regulation of organic compounds essential for stress acclimation. These solutes play fundamental roles in maintaining osmotic balance, stabilizing proteins, and protecting against oxidative damage [38].

The contents of soluble carbohydrates in the flowers were negatively affected by the isolated application of nicotinamide (T4) and its combinations with salicylic acid (T7) and with all three attenuators (T8), indicating a potential limitation in carbohydrate metabolism under these conditions. The lower carbohydrate content in plants exposed to high salinity may be attributed to ion accumulation, particularly Na^+^ and Cl^−^, which reduces the photosynthetic rate and CO_2_ fixation. This leads to a decrease in electron transfer in photosystem II, impairing the accumulation, distribution, and translocation of sugars in plant tissues [39]. Additionally, the rapid formation of ROS causes damage to macromolecules such as nucleic acids, proteins, carbohydrates, and lipids [40]. Conversely, the treatment with proline and salicylic acid (T5) maintained soluble carbohydrate levels similar to the control (T0) and the salt-stressed treatment without attenuators (T1), suggesting that this combination supports the maintenance of carbohydrate metabolism, which is crucial for stress acclimation [41]. The effect of proline on carbohydrate levels may be associated with the preservation of net photosynthesis in plants under salt stress, an essential process for the accumulation of this primary metabolite [18].

Salinity reduced the levels of free amino acids in the flowers, with the control (T0) showing the highest accumulation. Under stress conditions, treatments with nicotinamide alone (T4), combined with proline (T6), salicylic acid (T7), or all three attenuators (T8) resulted in slightly higher levels but still below those of the control. This may be attributed to the redirection of free amino acids toward the synthesis of proteins or other stress-related compounds, such as polyamines [1,15]. The slight increase observed in some treatments suggests a partial contribution to osmotic protection and metabolic regulation.

The combination of nicotinamide with salicylic acid (T7) resulted in the highest soluble protein levels, indicating a synergistic effect between these attenuators in promoting protein synthesis. This effect is likely due to the enhanced regulation of nitrogen metabolism and the activation of antioxidant enzymes [42]. The induction of multifunctional proteins, such as glutathione transferases, is considered an essential adaptive strategy to maintain redox homeostasis under adverse conditions [43].

Proline, a compatible solute, contributes to osmotic adjustment, scavenges ROS, regulates nutrient uptake, protects biological membranes and cellular organelles from oxidative damage, and enhances plant tolerance to salinity by increasing endogenous levels through exogenous application [44,45]. Proline, one of the most important compatible solutes under salt stress, accumulated in the flowers and leaves in response to the attenuators. The application of salicylic acid alone (T3) and in combination with nicotinamide (T7) resulted in the highest levels of free proline in the flowers, while the lowest values were observed in the treatment without attenuators (T1). The increase in proline levels across all treatments with attenuators confirms its central role in salt stress acclimation, assisting in the maintenance of cellular integrity and osmotic balance [37].

The results of this study highlight the negative impact of salt stress on the growth and biomass production of nasturtium, while the application of attenuators can mitigate these effects, depending on the compound and combination used. Salinity is known to trigger osmotic and ionic stress, impairing essential physiological processes such as nutrient absorption, water balance, and photosynthesis, ultimately leading to reduced vegetative and reproductive growth in plants [46]. However, the attenuators tested demonstrated varying degrees of effectiveness in offsetting these deleterious effects.

The greater plant height observed with the isolated use of salicylic acid (T3) can be attributed to its ability to regulate processes such as photosynthesis, ion homeostasis, and antioxidant signaling [17,20]. Salicylic acid is widely recognized for promoting the expression of genes involved in adaptive stress responses, including the synthesis of antioxidant enzymes and heat shock proteins, which protect cells against oxidative damage [47]. Conversely, the reduced height observed in the treatment with proline alone (T2) suggests that, under certain conditions, proline alone may not be sufficient to counterbalance the negative impacts of salt stress, possibly due to limitations in its capacity for metabolic and osmotic regulation.

The increase in the number of leaves observed in the treatment with nicotinamide combined with salicylic acid (T7) reflects a synergistic effect between these compounds, which may have enhanced photosynthetic efficiency and the redistribution of assimilates toward vegetative development [28,31]. Vitamins such as niacin are essential for the synthesis of coenzymes involved in biochemical reactions related to energy metabolism, while salicylic acid improves membrane stability and chloroplast functionality, boosting the plant’s photosynthetic capacity [20,21].

The higher flower production observed with salicylic acid (T3) and the combination of proline and nicotinamide (T6) indicates that these treatments promoted resource allocation to reproductive structures even under stress conditions [15,17]. Proline, as a compatible solute, may have contributed to maintaining cellular turgor and enzymatic stability [18], while nicotinamide, a precursor of NAD and NADP, functions as an essential cofactor in cellular and respiratory metabolism. It also supports energy transport, plant growth, and development through photosynthesis in the oxidative pentose phosphate pathway and mitochondrial metabolism [48], thereby enhancing floral induction. Conversely, the reduction in the number of floral buds with proline alone or combined with salicylic acid (T5) suggests that specific interactions between attenuators may generate antagonistic effects on reproductive development, possibly due to hormonal imbalances or energy limitations.

The increase in flower dry biomass observed with salicylic acid (T3) highlights its effectiveness in mitigating the effects of salt stress on reproductive organs. Salicylic acid may have contributed to the protection of photosynthesis and the redistribution of assimilates toward flower development, reducing the impacts of stress caused by excess salts [42]. The higher root dry biomass observed with salicylic acid alone (T3) and in combination with nicotinamide (T7) may be linked to improved water and nutrient uptake. Salicylic acid enhances root hair growth [49], facilitating water and nutrient absorption under abiotic stress conditions. Additionally, nicotinamide promotes cell elongation, reserve accumulation, and vegetative growth even under salinity by increasing indoleacetic acid (IAA) levels, which stimulate cell division and the plant’s metabolic activities [28,50].

The results of this study reinforce the effectiveness of attenuators such as salicylic acid, nicotinamide, and proline in mitigating the effects of salt stress in nasturtium. The differentiated performance of the treatments highlights the importance of specific combinations to maximize the benefits of these compounds, considering their interactions and complementary functions. These findings provide a solid foundation for developing more efficient management strategies in hydroponic systems under saline conditions, contributing to the sustainability of agricultural production in adverse environments.

## 4. Materials and Methods

### 4.1. Experimental Place, Experimental Structure, and Design

The experiment was conducted in a greenhouse at the Graduate Program in Agricultural Engineering of the Universidade Federal do Recôncavo da Bahia (12°40′19′′ S, 39°06′23′′ W, with an altitude of 220 m), located in Cruz das Almas, Bahia, Brazil. The study was conducted using a completely randomized design, combining two salinity levels (0 mM—control and 40 mM NaCl) with the application of three salt stress attenuators [salicylic acid—SA (1 mM), proline—Pro (10 mM), and nicotinamide—NAM (300 mg L^−1^)], applied individually and in combinations. In total, nine treatments were evaluated (Table 1), with four replications.

The experimental setup consisted of two types of hydroponic systems. The first, a nursery based on the Nutrient Film Technique (NFT), was constructed using corrugated PVC tiles with a 3% slope. This system included a reservoir with a 40 L capacity for the nutrient solution (NS) and a timer programmed to recirculate the solution for 15 min at 15 min intervals during the daytime (from 6:00 a.m. to 6:00 p.m.). The second hydroponic system, a Deep Flow Technique (DFT) known as floating, maintained the roots submerged in the NS, supported by Styrofoam boards placed in buckets with a 15 L capacity. This system was equipped with an aeration system activated by a timer, configured to operate for 15 min with 2 h intervals during both the daytime (6:00 a.m. to 6:00 p.m.) and nighttime (6:00 p.m. to 6:00 a.m.).

### 4.2. Growth Conditions

In the present study, the seeds of *T. majus* var. ‘Anã Sortida’ (ISLA Sementes, Porto Alegre, RS, Brazil) were sown in phenolic foam with 5 mm perforations, previously sterilized in a 5% sodium hypochlorite solution, and maintained under adequate humidity conditions for germination. The experiment began with the sowing of one seed per phenolic foam cell. Eight days after sowing (DAS), the seedlings were transferred to the nursery and kept in a nutrient solution (NS) formulated based on the recommendations of Furlani et al. [51] at 50% concentration for four days. At 12 DAS, the seedlings were transferred to the floating hydroponic system and subjected to the pre-defined salinity treatments. The nutrient solution was prepared following an adaptation of the recommendations by Furlani et al. [51] at 100% concentration, and salinity was adjusted to 40 mM by adding sodium chloride (NaCl). Preliminary tests (unpublished data) were conducted under the same experimental conditions prior to setting up the experiment described in this paper to determine an NaCl concentration that would cause moderate salt stress in the plants. The concentration of 40 mM NaCl was found to be the most suitable according to these preliminary tests.

Nine days after transplantation (DAT), foliar application of the attenuators salicylic acid (SA—1 mM, Sigma Aldrich, São Paulo, Brazil), proline (Pro—10 mM, Synth, Diadema, SP, Brazil), and nicotinamide (NAM—300 mg L^−1^, Synth, Diadema, SP, Brazil) was initiated. The foliar treatment was performed weekly, always in the early morning, using manual sprayers equipped with drop regulators. To enhance the adhesion of the attenuators to the leaf surface, Tween 20 (0.05%) was added to each solution applied. A total of 550 mL of each treatment was applied to each plant until the end of the experiment, with this amount being divided into six applications throughout the cultivation period. At 15 DAT, saline water replenishments (40 mM NaCl) were initiated, except for the control treatment. These replenishments were performed every five days, always in the late afternoon. At the same intervals, measurements of the electrical conductivity (EC) and pH of the nutrient solution were performed.

### 4.3. Variables Analyzed

#### 4.3.1. Gas Exchange

Gas exchange variables were measured using an infrared gas analyzer (IRGA, LICOR, Lincoln, NE, USA). Measurements were conducted between 8:00 and 10:00 a.m. under controlled conditions, with artificial light set to 1000 µmol photons m^−2^ s^−1^, ambient CO_2_ concentration, and temperature. The evaluated variables included stomatal conductance (*gs*, mol H_2_O m^−2^ s^−1^), net photosynthesis rate (*A*, µmol CO_2_ m^−2^ s^−1^), transpiration rate (*E*, mmol H_2_O m^−2^ s^−1^), intercellular CO_2_ concentration (*Ci*, µmol CO_2_ mol air^−1^), instantaneous water-use efficiency (WUE = *A*/*E*), intrinsic water-use efficiency (iWUE = *A*/*gs*), and *Ci*/*Ca* ratio. These measurements were performed on fully expanded leaves from the middle third of the plants.

#### 4.3.2. Photosynthetic Pigments

For the determination of the chlorophyll a, chlorophyll b, and carotenoid contents, 20 mg of lyophilized material was used. The material was transferred to test tubes, where leaf extracts were collected, and 3 mL of 80% acetone was added. The tubes were stored under refrigeration (4 °C) in the dark for 24 h to ensure pigment stability. The samples were centrifuged at 14,000× *g* for 15 min, and the supernatants were collected. The contents of chlorophylls and carotenoids were determined by spectrophotometry, with readings performed at wavelengths of 664.1 nm, 648.6 nm, and 470 nm, following the methodology described by Lichtenthaler and Buschmann [52]. Additionally, total chlorophyll was calculated as the sum of chlorophyll a and b.

#### 4.3.3. Inorganic Solutes

To prepare the samples for inorganic solute analysis, flowers and leaves previously dried in an oven were used. After drying, the material was ground, and 0.1 g of each sample was weighed and transferred to test tubes. Subsequently, 10 mL of deionized water was added to each tube. The samples were then placed in a thermostatic bath at 100 °C for 1 h. After heating, the solutions were filtered and prepared for subsequent analyses. The contents of Na^+^ and K^+^ were determined using a flame photometer (model Q498M2, QUIMIS, Diadema, SP, Brazil) according to the method described by Silva et al. [53]. The Cl^−^ content was determined by spectrophotometry using a spectrophotometer (model 2000 UV, BEL PHOTONICS, Piracicaba, SP, Brazil) based on the methodology of Silva et al. [54].

#### 4.3.4. Organic Solutes

For the determination of organic solutes, three leaves and three flowers were collected, separately stored in bags, and subjected to lyophilization. After the lyophilization process, the samples underwent a two-step extraction protocol. Initially, 0.1 g of the lyophilized material was weighed into microcentrifuge tubes (Eppendorf), and 1.5 mL of 80% ethanol was added. The samples were centrifuged at 14,000× *g* for 15 min, and the supernatants were collected. Subsequently, 1.5 mL of 80% ethanol was added again to the residue, and the same procedure was repeated.

The quantification of soluble carbohydrates was performed according to the method described by Dubois et al. [55], using 0.5 mL of the extract and the phenol–sulfuric acid method, with colorimetric reading at 490 nm and D-(+)-glucose as the standard. The determination of free proline was based on the method of Bates et al. [56], using 0.5 mL of the extract and ninhydrin reagent, with a colorimetric reading at 520 nm and pure proline as the standard.

Free amino acids were quantified according to the method of Yemm and Cocking [57], using 0.5 mL of the extract and ninhydrin reagent, with a colorimetric reading at 570 nm and pure L-leucine as the standard. Soluble proteins were determined using the Bradford method [58], employing 0.1 mL of the extract and the dye-binding method, with a reading at 595 nm and bovine serum albumin as the standard.

#### 4.3.5. Growth and Production

The biometric analyses included measuring plant height (PH), counting the number of leaves, the number of flowers, and the number of flower buds. The production analyses involved determining the dry mass of the stem, leaves, roots, and flowers. All collections were carried out at the end of the experimental cycle, 51 days after transplantation (DAT). To obtain the data, the plants were harvested and separated into their different components. The materials were stored in paper bags and then dried in a forced-air circulation oven at 65 °C for 72 h to determine the dry masses.

### 4.4. Statistical Analysis

The data obtained were subjected to analysis of variance (ANOVA) using the F-test. All data were tested for normality (Shapiro–Wilk test) and homogeneity of variance (Bartlett’s test). Treatment means were compared using the Scott–Knott test (*p* ≤ 0.05). All statistical analyses were performed using the R software version 4.3.1 [59].

## 5. Conclusions

The combination of nicotinamide, salicylic acid, and proline positively influenced *Tropaeolum majus* by improving physiological and biochemical responses under salt stress, including enhanced gas exchange parameters such as stomatal conductance, transpiration, and intercellular CO_2_ concentration. This treatment also stabilized photosynthetic pigments, promoted osmotic balance by increasing proline and soluble sugar levels, and reduced the detrimental effects of ion accumulation and oxidative stress. Additionally, the combination enhanced the stability of chloroplasts and protected cellular structures, resulting in better overall plant growth and biomass allocation, particularly in reproductive tissues.

## Figures and Tables

**Table 1:** Details of treatments for growing nasturtium.

Treatment	Description
T0	0 mM NaCl (control)
T1	40 mM NaCl
T2	40 mM NaCl + Pro
T3	40 mM NaCl + SA
T4	40 mM NaCl + NAM
T5	40 mM NaCl + Pro + SA
T6	40 mM NaCl + Pro + NAM
T7	40 mM NaCl + NAM + SA
T8	40 mM NaCl + NAM + SA + Pro

## Data Availability

All data produced and/or analyzed in this study are included in the manuscript. The corresponding authors are available to provide additional data and materials upon reasonable request.
